# Early microglial activation and peripheral inflammation in dementia with Lewy bodies

**DOI:** 10.1093/brain/awy265

**Published:** 2018-11-06

**Authors:** Ajenthan Surendranathan, Li Su, Elijah Mak, Luca Passamonti, Young T Hong, Robert Arnold, Patricia Vázquez Rodríguez, William R Bevan-Jones, Susannah A E Brain, Tim D Fryer, Franklin I Aigbirhio, James B Rowe, John T O’Brien

**Affiliations:** 1 Department of Psychiatry, University of Cambridge, Cambridge, UK; 2 Sino-Britain Centre for Cognition and Ageing Research, Faculty of Psychology, Southwest University, Chongqing, China; 3 Department of Clinical Neurosciences, University of Cambridge, Cambridge, UK; 4 Wolfson Brain Imaging Centre, University of Cambridge, Cambridge, UK; 5 Royal Berkshire NHS Foundation Trust, Reading, UK

**Keywords:** inflammation, dementia with Lewy bodies, cytokines, microglia, neurodegeneration

## Abstract

Inflammation is increasingly recognized as part of the pathology of neurodegenerative conditions such as Alzheimer’s disease and Parkinson’s disease, but its role in dementia with Lewy bodies remains unclear. Using multimodal imaging and peripheral cytokine analysis, we therefore investigated central and peripheral inflammation in this common form of dementia. Nineteen participants with probable dementia with Lewy bodies and 16 similarly aged controls underwent 3 T MRI and PET imaging with ^11^C-PK11195, a marker of microglial activation *in vivo*. Peripheral blood inflammatory cytokines were also measured in all subjects, as well as in an additional 10 controls, using the Mesoscale Human Cytokine 36 plex panel and additional assays for high sensitivity c-reactive protein, tumour necrosis factor receptor 1, IL-34, YKL-40 (chitinase-3-like protein 1) and colony stimulating factor 1. To test for the presence of *in vivo* amyloid, ^11^C-Pittsburgh compound B PET imaging was also performed in 16 of the dementia with Lewy body participants. Microglial activation was elevated in dementia with Lewy bodies subjects with mild disease when compared to those with moderate/severe impairment, where disease severity was indexed by cognitive performance on the revised Addenbrooke’s Cognitive Examination. In patients, strong correlations were found between cognitive performance and ^11^C-PK11195 non-displaceable binding potential in several regions including the caudate nucleus (R = 0.83, *P =* 0.00008) and cuneus (R = 0.77, *P* = 0.0005). Several inflammatory cytokines were altered in the patients compared to controls, with elevated macrophage inflammatory protein-3 (*P* = 0.001), IL-17A (*P* = 0.008) and IL-2 (*P* = 0.046) and reduced IL-8 (*P* = 0.024). There was no correlation between cortical ^11^C-Pittsburgh compound B standardized uptake value ratio and clinical features, regional ^11^C-PK11195 binding or peripheral cytokine levels. Nor was there any regional correlation between ^11^C-PK11195 non-displaceable binding potentials and ^11^C-Pittsburgh compound B standardized uptake value ratios. Our findings provide evidence for both central and peripheral inflammatory changes in dementia with Lewy bodies, with microglial activation occurring early in the disease in key regions known to be associated with pathology, before declining as cognition declines. Raised peripheral cytokines associated with T cell function further suggest a role for the adaptive immune system in the pathogenesis of the disease.

## Teaser

The role of inflammation in dementia with Lewy bodies is unclear. Surendranathan *et al.* report that microglial activation is elevated in mild disease, before decreasing as cognition declines. Inflammatory markers in the blood are also altered compared to control subjects, suggesting that inflammation could be targeted early in the disease.

## Introduction

Dementia with Lewy bodies (DLB) is the second commonest degenerative form of dementia in older people (Fujimi *et al.*, 2008; Jellinger and Attems, 2011) and is characterized by α-synuclein protein aggregations in the form of Lewy bodies within neurons and Lewy neurites. Neuroinflammation is increasingly considered as a contributor to dementia pathogenesis (Amor *et al.*, 2014), and a potential target for novel disease-modifying therapeutic strategies. α-Synuclein aggregates are reported to interact with a range of components of the immune system including microglia (Surendranathan *et al.*, 2015), potentially providing the substrate for an inflammatory response.

In diseases closely related to DLB, through shared or co-morbid pathology, such as Parkinson’s disease (Dobbs *et al.*, 1999; Imamura *et al.*, 2003; Wahner *et al.*, 2007; Brochard *et al.*, 2009; Hamza *et al.*, 2010), and Alzheimer’s disease (Lee *et al.*, 2010; McGeer and McGeer, 2013; Morales *et al.*, 2014; Latta *et al.*, 2015; Lai *et al.*, 2017; Passamonti *et al.*, 2018), inflammation has been identified with a range of methodologies, including pathological, genetic, epidemiological and with cytokine assessment.

Recent studies suggest inflammation occurs early. Inflammation is reported in the early stages of Alzheimer’s disease as well as in mild cognitive impairment even before the onset of dementia (Okello *et al.*, 2009; Hamelin *et al.*, 2016). Similarly, in Parkinson’s disease, PET imaging shows early inflammation *in vivo* in the brainstem before extending cortically as the disease progresses. By the onset of dementia, increased microglial activation appears to be widespread (Ouchi *et al.*, 2005; Gerhard *et al.*, 2006; Fan *et al.*, 2015). Patients affected by rapid eye movement (REM) sleep behaviour disorder, which is now recognized as a prodromal stage of synucleinopathies (Högl *et al.*, 2018), show elevated microglial activation in the substantia nigra (Stokholm *et al.*, 2017).

Direct evidence of inflammation in Lewy body disease is growing, with elevated microglial activation identified at post-mortem (Togo *et al.*, 2001) and on PET in one small case series (Iannaccone *et al.*, 2013). Exploratory next generation gene sequencing indicates an inflammatory component in DLB pathology, with specific antigen presentation alleles (HLA-DPA1/DPB1) increasing risk (Peuralinna *et al.*, 2015). Elevated interleukins have also been reported in prodromal DLB, although not the established disease (King *et al.*, 2017). In Parkinson’s disease dementia, another form of dementia associated with Lewy bodies, a rise in c-reactive protein has been identified, after the onset of dementia (Song *et al.*, 2013).

Inflammation represents a potential means of modifying disease progression in dementia. Whether central or peripheral, therapies attenuating inflammation may be able to slow or even halt progressive neurodegeneration. Anti-inflammatory treatments already exist, meaning that in contrast to the disappointing results thus far for the discovery of therapies targeting protein accumulation in dementia, therapies targeting inflammation could be brought into clinical practice more quickly. The identification of inflammation as an early part of the disease process would increase its usefulness as a target with treatment then possible in the prodromal phase.

However, more definitive evidence of peripheral and central inflammation *in vivo* in DLB is needed, as well as further evidence of the stage(s) in the disease process at which inflammation occurs, essential information for planning future therapeutic studies. A deeper understanding of the *in vivo* relationship between central and peripheral inflammation in the same patients is also required, to better elicit the role of inflammation in the pathophysiology of the disease and its effect on the clinical syndrome.

We hypothesized that patients with DLB would have increased central and peripheral inflammatory changes when compared to controls and that central inflammation would correlate with peripheral inflammation. To assess for these differences, we undertook PET imaging with ^11^C-PK11195 (PK11195), a marker of microglial activation *in vivo* within the brain, and tested for peripheral inflammatory cytokines in patients with DLB and healthy controls. We predicted that these changes would vary according to disease severity, with more pronounced changes early in disease as has been found in both Alzheimer’s disease and Parkinson’s disease. Accordingly, we assessed cognitive and motor performance in each subject for comparison with any central or peripheral inflammatory changes.

Finally, in view of the concurrent amyloid-β pathology found in many DLB patients (Colom-Cadena *et al.*, 2013), we also tested for concomitant amyloid protein deposition using ^11^C-Pittsburgh compound B (PIB) PET and assessed whether amyloid load correlated with inflammation centrally or peripherally.

## Materials and methods

### Participants

All participants were aged over 50 years and had sufficient proficiency in English for cognitive testing. Nineteen patients with ‘probable’ DLB as defined by both 2005 and 2017 consensus criteria (McKeith *et al.*, 2005, 2017), and 26 age- and gender-matched healthy controls were recruited. Exclusion criteria were: (i) acute infection; (ii) a contra-indication to MRI, or a history of any of the following: (iii) major psychiatric disorder (e.g. major depression); (iv) neurological disorder (except a diagnosis of DLB in DLB subjects); (v) head injury; or (vi) systemic inflammatory disorder (e.g. systemic lupus erythematosus, rheumatoid arthritis or Crohn’s disease).

Patients were identified from the specialist memory clinic at the Cambridge University Hospitals NHS Trust, other local memory clinics, from the Dementias and Neurodegenerative Diseases Research Network (DeNDRoN) volunteer registers or the Join Dementia Research platform (https://www.joindementiaresearch.nihr.ac.uk). Healthy controls were recruited via DeNDRoN or Join Dementia Research as well as from spouses and partners of participants. Informed written consent was obtained from participants and their designated informants in accordance with the Declaration of Helsinki. The study received a favourable opinion from the East of England (Cambridge Central Research) Ethics Committee (reference: 13/EE/0104). The methodology has been previously published in the study protocol paper (Bevan-Jones *et al.*, 2017), but is briefly described below.

### Clinical assessments

All participants underwent an initial assessment that included neuropsychological and cognitive testing [including Mini Mental State Examination (MMSE) and Addenbrooke’s Cognitive Examination-Revised (ACE-R)], severity of parkinsonism [Unified Parkinson’s Disease Rating Scale part III - motor (UPDRS)] and demographic measures.

### MRI and PET imaging

All participants underwent MRI on a 3 T Siemens Magnetom Tim Trio, Verio or Skyra scanner. Each MPRAGE (magnetization-prepared rapid acquisition gradient-echo) T_1_-weighted sequence was non-rigidly registered to the ICBM2009a template brain using ANTS (http://www.picsl.upenn.edu/ANTS/) and the inverse transform was applied to a modified Hammers atlas (resliced from MNI152 to ICBM2009a space) to bring the regions of interest to subject MRI space, to which the PET data described below were co-registered.

Nineteen DLB and 16 control group participants underwent PK11195 PET imaging to assess the extent and distribution of microglial activation, using a GE Advance PET scanner (GE Healthcare) or a GE Discovery 690 PET/CT, with attenuation correction provided by a transmission scan or a low dose CT scan, respectively. The emission protocol for PK11195 was 75 min of dynamic imaging consisting of 55 frames starting concurrently with a 500 MBq PK11195 injection. Binding in each region of interest was quantified using non-displaceable binding potential (BP_ND_) determined with a simplified reference tissue model incorporating vascular binding correction and reference region time activity curve estimation from supervised cluster analysis using four kinetic classes (Yaqub *et al.*, 2012). Regional BP_ND_ was corrected for CSF contamination through division of the region of interest time activity curve with the mean region of interest fraction of grey and white matter, using SPM8 (www.fil.ion.ucl.ac.uk/spm/software/spm8) probability maps smoothed to match the PET spatial resolution. Sixteen of the DLB participants also underwent PIB PET imaging, with 550 MBq of PIB injected as a bolus and imaging performed for 30 min starting at 40 min post-injection. PIB data were quantified using standardized uptake value ratio (SUVR) by dividing the mean CSF-corrected radioactivity concentration in each Hammers atlas region of interest by the corresponding mean CSF-corrected radioactivity concentration in the cerebellar grey matter reference tissue region of interest. Participants were considered amyloid positive if the average SUVR value across the cortical regions of interest was > 1.4, a cut-off that has been identified as having the highest specificity for amyloid pathology in a recent autopsy study (Villeneuve *et al.*, 2015). While a lower value of 1.2 was found to be more sensitive for early amyloid pathology, this is of less importance in this study, as we have proceeded to carry out correlation analysis using PIB SUVR in all participants regardless of amyloid status, in order to investigate the effect of amyloid load.

### Cytokine assessments

Blood samples were obtained from all participants, allowed to clot for at least 30 min, centrifuged to isolate serum, then aliquoted and stored at 70°C until cytokine analysis. Assays were carried out by the Core Biochemical Assay Laboratory, Cambridge University Hospital, using the MesoScale Discovery V-Plex Human Cytokine 36 plex panel and five additional cytokine assays: high sensitivity c-reactive protein (using Siemens Dimension EXL autoanalyser), tumour necrosis factor receptor 1, interleukin-34 (IL-34), YKL-40 (chitinase-3-like protein 1), all using Bio-Techne R&D Systems kit, and colony stimulating factor 1 using the electrochemiluminescence immunoassay from MesoScale Discovery. Dilutions were made in accordance with manufacturer recommendations. Each assay was performed in duplicate, with the mean taken for the purposes of analysis. Further details of the cytokine assays can be found in Supplementary Table 1: details of cytokine assays.

### Statistical analysis

Statistical analysis was completed using IBM SPSS Statistics software (version 25) and support vector machine (SVM) analysis carried out with R: R Foundation for Statistical Computing, Vienna, Austria (https://www.R-project.org/).

Demographics were compared using Student’s *t-*test for continuous variables and chi-squared test or Fisher’s exact test for categorical variables. To compare cytokine levels between the DLB and cytokine control group, a repeated measures general linear model was used to test for the effect of group as well as a group × cytokine interaction, with age and gender included as covariates of no interest. The majority of the cytokine assay results were positively skewed, hence all cytokine measurements were transformed with log10(*x* + 1) prior to analysis to improve normality for the general linear model. PK11195 BP_ND_ for the DLB and PET control groups was also compared using a repeated measures general linear model, with age, gender and education included as covariates of no interest.

To study whether cytokine profiles or PK11195 binding in regions of interest could differentiate subjects according to group further, we used an SVM with feature selection to select the best variables from these datasets and identify the highest rate of accuracies that could be obtained for classification into groups. We trained the SVM model with leave-one-out cross-validation and a linear kernel tuned to provide the optimum balance between a wide margin between support vectors in the hyperplane and a small number of misclassified data points. Both leave-one-out cross-validation and a linear kernel (compared to a radial kernel) (Hua *et al.*, 2005) reduce the risk of over-fitting the data in the training phase. Application of the SVM across different training group partition sizes, where each subject was randomly allocated to testing or training, identified: (i) the training and testing split that provided the highest accuracies; and (ii) an order of influence of each variable as support vectors.

Next, we performed feature selection similar to that previously used to identify optimum blood biomarker panels in Alzheimer’s disease (Long *et al.*, 2016). Features were individually added in order of increasing influence to create an enlarging panel of variables. For each panel, the SVM was repeated 5000 times, each with randomly allocated training groups from the full list of sample subjects, to obtain the mean accuracy for classification into groups. Once the panel with the highest accuracy was obtained, features were further selected within this subset based on changes in accuracy following their removal from the panel, to identify a set of features that recorded the peak accuracy.

Correlations between clinical factors (disease duration and disease severity measured through ACE-R and UPDRS), regional PK11195 BP_ND_, amyloid SUVR, and cytokine levels were assessed with Pearson partial correlation, with age, gender and education as covariates of no interest. To correct for multiple comparisons, the Benjamini-Hochberg false detection rate method was applied, with an alpha of 0.05.

### Data availability

The data that support the findings of this study are available from the senior author (J.T.O.), upon reasonable request. The underlying PET and magnetic resonance images will be made available via the DPUK portal (https://portal.dementiasplatform.uk), for academic and non-commercial purposes, once a parallel study based on the same original data is completed.

## Results

There were no differences in age or gender between the DLB group and the two control groups [both the smaller PK11195 cohort and the expanded cytokine cohort (Table 1)], though the DLB group had fewer years of formal education than the two control groups. As expected DLB participants had lower cognitive scores as measured by the ACE-R and MMSE compared to the control participants.

**Table 1:** Participant demographics

	**DLB (*n =* 19)**	**Control group**		**Group difference DLB versus control group**	
		**PK11195 PET imaging (*n =* 16)**	**Cytokines (*n =* 26)**	**PK11195 PET**	**Cytokines**
Gender, males/females	15/4	8/8	15/11	*P* = 0.09	*P* = 0.20
Age, years, mean ± SD	73.0 ± 6.1	70.0 ± 6.5	69.9 ± 6.4	t = 1.4; *P* = 0.17	t = 1.6; *P* = 0.11
Education, years, mean ± SD	11.7 ± 1.9	14.1 ± 3.0	14.7 ± 2.8	t = −2.9; *P* = 0.007	t = −4.1; *P* < 0.001
MMSE scores, mean ± SD	21.9 ± 4.5	28.9 ± 1.1	29.1 ± 0.9	t = −6.7; *P* < 0.001	t = −6.9; *P* < 0.001
ACE-R scores, mean ± SD	65.7 ± 12.9	92.5 ± 5.6	94.0 ± 5.0	t = −8.2; *P* < 0.001	t = −9.1; *P* < 0.001
UPDRS scores, mean ± SD	32.5 ± 20.6	N/A	N/A	-	-
Disease duration, years, mean ± SD	4.2 ± 2.7	N/A	N/A	-	-
^11^C-PK11195 PET scan	19	16	16	-	-
^11^C-PiB PET scan	16	0	0	-	-

The control groups for PET and cytokines (the latter consisting of 10 additional participants) were each matched with the DLB group for gender and age.

### PK11195 PET

In view of our prediction of differential inflammation between early and late stages of disease and the marked variation in severity as highlighted by the standard deviations in ACE-R scores (13) and UPDRS scores (21), the DLB group was split using the median ACE-R score, resulting in nine ‘mild’ cases with an ACE-R of > 65 (mild DLB group) and 10 ‘moderate/severe’ cases with an ACE-R of ≤ 65 (moderate/severe DLB group), reflecting levels of cognitive impairment at the time of their PK11195 scan.

All three groups (mild and moderate/severe DLB, plus controls) were matched for gender and age (Supplementary Table 2). Education was also matched between the mild and moderate/severe DLB groups, but both groups’ years of education were lower than controls. As expected, MMSE and ACE-R scores were significantly different between each of the three groups. Disease duration and UPDRS scores were higher in the moderate/severe DLB group than the mild DLB group, but this was not statistically significant.

Repeated measures general linear model analysis of these three groups, with age, gender and education as covariates, showed a significant main effect of group [*F*(2,29) = 5.88, *P* = 0.007], and a main effect of region [*F*(8,231) = 2.1, *P* = 0.04], but no group × region interaction [*F*(16,231) = 1.35, *P* = 0.171; Greenhouse-Geisser corrections (ε = 0.198) to the degrees of freedom were required as sphericity was violated (*P* < 0.001)]. Pairwise comparisons showed the main group effect was due to a significant difference between the mild and moderate/severe DLB groups (*P* = 0.006).

**Figure 1:**
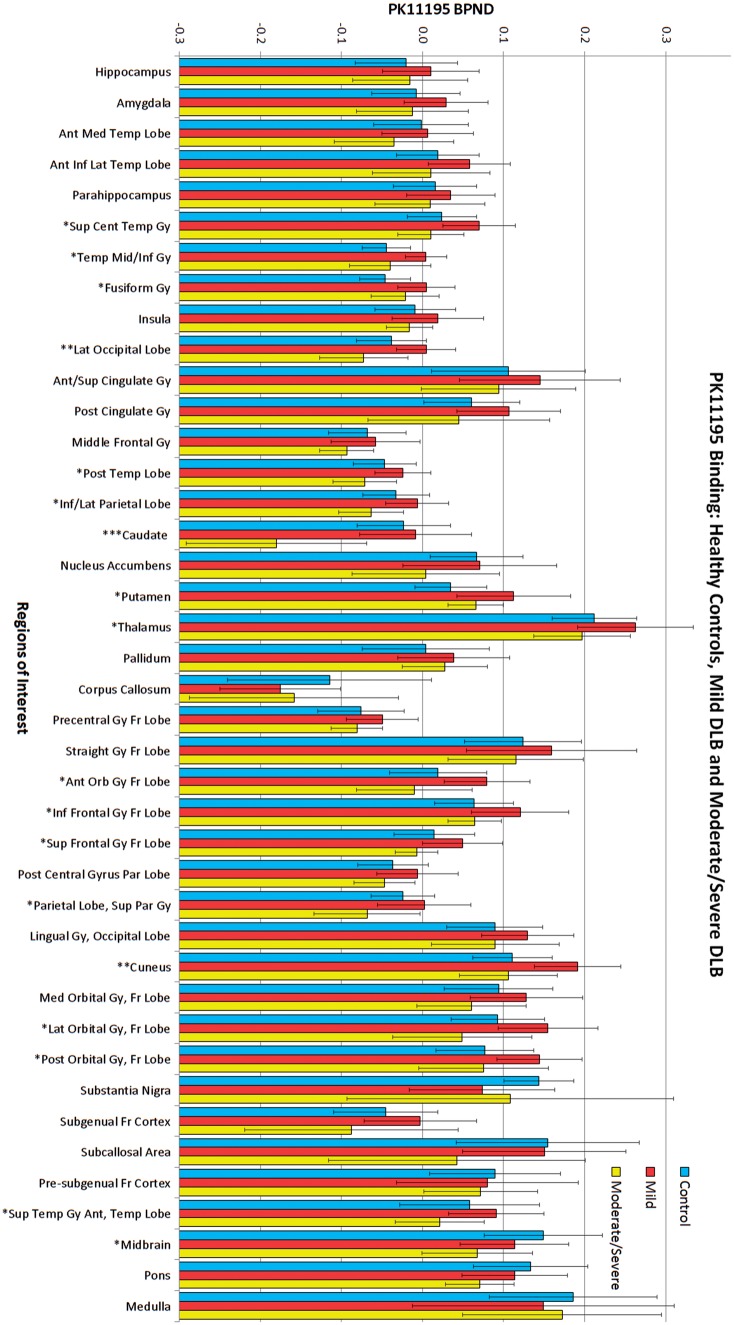
**Group differences in PK11195 BP_ND_.** PK11195 BP_ND_ between controls, mild and moderate/severe DLB; significant differences were found in 18 regions. Significance: ***P < 0.001, **P < 0.01, *P < 0.05. Error bars represent standard deviation. ant = anterior; cent = central; gy = gyrus; inf = inferior; lat = lateral; med = medial; orb = orbital; par = parietal; post = posterior; sup = superior; temp = temporal.

*Post hoc* ANCOVAs with the same covariates found 18 of 41 regions to be significantly different between all three groups: caudate nucleus, cuneus, lateral occipital lobe, inferior frontal gyrus, middle and inferior temporal gyrus, central superior temporal gyrus, lateral orbital gyrus, anterior orbital gyrus, superior frontal gyrus, inferolateral parietal lobe, superior parietal gyrus, posterior temporal lobe, posterior orbital gyrus, putamen, anterior superior temporal gyrus, fusiform gyrus, midbrain and thalamus. The caudate nucleus [*F*(29,2) = 12.702, *P* = 0.0001] showed the highest level of significance (Fig. 1).

Pairwise comparisons from the ANCOVAs found 14 individual regions had significant differences in binding between DLB groups, all with mean BP_ND_ higher in the mild DLB group: superior and middle/inferior temporal gyri, lateral occipital lobe, posterior temporal lobe, inferior lateral parietal lobe, caudate, thalamus, anterior orbital gyrus, inferior frontal gyrus, superior frontal gyrus, superior parietal gyrus, cuneus, lateral orbital gyrus, and anterior superior temporal gyrus.

In addition, when comparing each DLB group with controls: 33 of 41 regions showed higher binding in the mild DLB group (an exact sign test used to compare the differences found a significant increase in PK11195 BP_ND_ in the mild DLB group compared to controls, *P* = 0.00004), with five significantly higher (inferior and medial temporal gyrus, fusiform gyrus, putamen, inferior frontal gyrus and cuneus) in the ANCOVA. Thirty-three of 41 regions showed higher binding in the control group than the moderate/severe DLB group (with an exact sign test finding a significant increase in the control group, *P* = 0.00004), but only the caudate nucleus was significantly higher.

Peak accuracy for the classification of DLB subjects from controls using the SVM model, based on PK11195 binding in regions of interest, was recorded at 75% (sensitivity of 68% and specificity of 84%) with the following five regions being the best for separating groups: caudate, putamen, midbrain, nucleus accumbens, and inferior frontal gyrus. For classifying mild from moderate/severe DLB subjects, the peak accuracy was 83% (sensitivity 75% and specificity 89%), with the cuneus, lateral occipital lobe, caudate, superior frontal gyrus, anterior superior temporal gyrus and anterior orbital gyrus best for separating the subgroups. BP_ND_ values were adjusted for age, gender and education prior to analysis by SVM.

### Cytokine analysis

Forty-one cytokines were assayed. Nine were removed from the analysis as follows. In three assays (IL-34, IL-23, IL-1β) there were no cytokines detected in any sample. In three further assays (IL-17A Gen B assay, IL-21, IL-31), between one and three samples had detectable levels—all within DLB subjects. For a further three assays less than a third of subjects in both groups had detectable levels (IL-1α, IL-5 and IL-4). Additionally, in two subjects there was a >20% variation when the assay was duplicated (one DLB IL-17A assay and one control IL-12p70 assay), hence the mean for the group was substituted. Where a cytokine assay result was below the detectable threshold, zero was substituted in as the result.

The repeated measures general linear model found no main effect of group [*F*(1,41) = 0.24, *P* = 0.63], however a significant group × cytokine interaction was found [*F*(12,500) = 1.92, *P* = 0.029 following Greenhouse-Geisser correction (ε = 0.39), as Mauchly’s test of sphericity was significant (*P* < 0.001) indicating that variances of the differences were not equal]. *Post hoc* ANCOVAs of each cytokine, with age and gender as covariates, showed that macrophage inflammatory protein 3α (MIP-3α) [*F*(41,1) = 13.29, *P* = 0.001], IL-17A [*F*(41,1) = 7.75, *P* = 0.008], and IL-2 [*F*(41,1) = 4.23, *P* = 0.046] were higher in DLB and that IL-8 [*F*(41,1) = 5.46, *P* = 0.024] was lower (see Table 2 for remaining results).

**Table 2:** Cytokine results

**Cytokine**	**Control participants**		**DLB participants**		**Log10 (*x* + 1) transform**		**% Difference in means**
	**Mean**	**SE**	**Mean**	**SE**	**F**	***P*-value**	
IL-12p70	0.228	0.229	6.06	25.7	0.732	0.397	2553
IL-2	0.140	0.218	0.496	0.637	4.234	0.046*	254
IL-22	0.907	0.805	1.91	3.62	0.867	0.357	111
IL-17A	3.04	1.58	5.67	6.19	7.747	0.008*	87
MIP-3a	3.09	2.59	5.74	3.27	13.298	0.001**	86
YKL-40	43034	28357	64150	46616	1.821	0.185	49
IP10	324	147	434	368	0.976	0.329	34
IL-12	151	119	174	90.6	1.894	0.176	15
TNFR1 (CD120a)	3186	949	3527	737	1.446	0.236	11
TNF α	2.92	1.02	3.16	0.99	0.033	0.856	9
IL-7	17.5	5.55	18.7	8.55	0.139	0.711	7
MCSF1	358	319	380	181	0.720	0.401	6
TNF-β	0.367	0.139	0.385	0.213	0.061	0.807	5
IL-27	2590	1421	2615	934	0.042	0.839	1
IL-6	0.975	0.704	0.974	0.531	0.163	0.689	0
IL-16	211	64.0	209	79.3	0.874	0.355	−1
IL-15	2.46	0.812	2.41	0.325	0.033	0.856	−2
hsCRP, mg/l	3.19	5.05	3.10	3.79	0.001	0.981	−3
GM-CSF	0.588	0.371	0.567	0.246	0.010	0.920	−3
MCP-1	282	100	268	81.3	0.004	0.951	−5
Eotaxin	181	52.4	171	83.0	1.726	0.196	−6
MIP1a	17.1	9.08	15.7	4.78	0.812	0.373	−8
MIP1b	130	74.6	119	35.8	0.158	0.693	−8
IL-10	0.463	1.10	0.420	0.281	0.550	0.463	−9
IFNγ	11.4	8.19	10.2	7.26	0.503	0.482	−10
MCP-4	197	59.4	175	58.8	1.498	0.228	−11
Eotaxin 3	21.0	7.51	18.6	5.90	2.255	0.141	−11
IL-13	0.594	0.749	0.512	0.697	0.723	0.400	−14
TARC	320	241	272	146	0.094	0.761	−15
IL-8	11.9	7.75	8.83	2.44	5.455	0.024*	−26
VEGF	178	115	116	56.3	1.455	0.235	−35
MDC	1644	2290	1018	161	0.899	0.349	−38

Results of *post hoc* ANCOVAs with age and gender as covariates of no interest (significance: *P < 0.05, **P < 0.005). Cytokines are presented in pg/ml except where stated, and are ordered according to differences in means between groups, with those cytokines highest in the DLB participants at the top. GM-CSF = granulocyte-macrophage colony-stimulating factor; hsCRP = high sensitivity c-reactive protein; IFN = interferon; IP-10 = interferon gamma-induced protein 10; MCP = monocyte chemotactic protein; MCSF = macrophage colony-stimulating factor; MDC = macrophage derived chemokine; MIP = macrophage inflammatory protein; TARC = thymus- and activation-regulated chemokine; TNF = tumour necrosis factor; TNFR = tumour necrosis factor receptor; VEGF = vascular endothelial growth factor.

With the SVM model, peak accuracy for the classification of subjects based on cytokines was recorded at 81% (sensitivity of 71% and specificity of 87% in classifying DLB subjects correctly). MIP-3α, IL-8, IL-2, IL-13, vascular endothelial growth factor, YKL-40 and IL-16 made up this discriminatory panel of cytokines. Cytokines were adjusted for age and gender prior to analysis by SVM.

### Amyloid status

Thirteen of 16 ‘probable’ DLB subjects who underwent PIB imaging, had SUVR > 1.4, indicating positive amyloid status.

### Correlation analysis in subjects with dementia with Lewy bodies

Pearson’s correlations were carried out between the clinical features (ACE-R score, UPDRS score and disease duration) and cortical PIB SUVR, together with log transformed cytokines and PK11195 BP_ND_ values in regions of interest. Regional correlations were also carried out between PK11195 BP_ND_ and PIB SUVR. Cytokines significantly different in DLB as identified using the repeated measures general linear model were selected for correlation, in addition to the 10 regions with the highest significant differences in PK11195 BP_ND_ from the repeated measures general linear model of the three groups.

**Figure 2:**
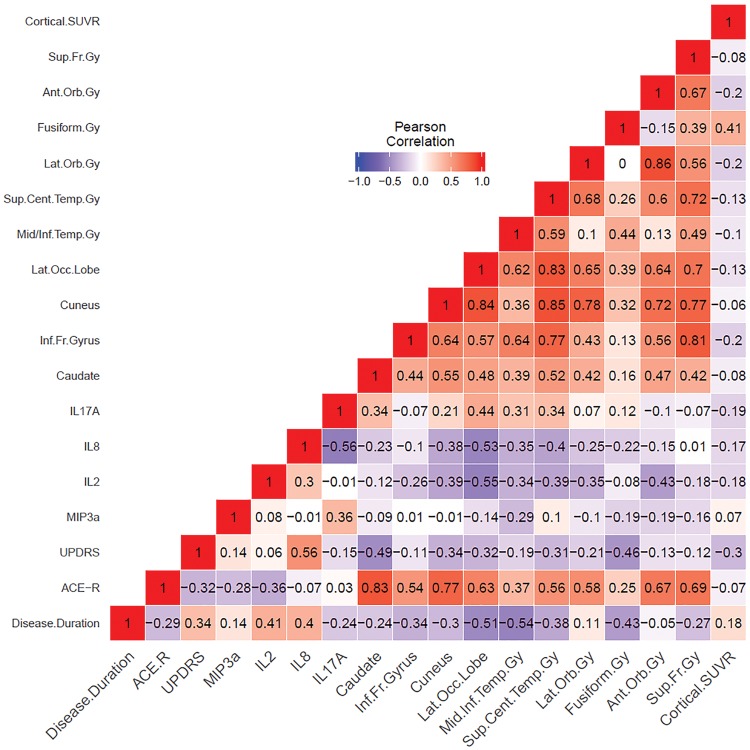
**Clinical, imaging and cytokine correlations.** Pearson’s partial correlations within the DLB group between clinical features, cytokines (log transformed) and PK11195 binding regions identified to have the greatest significant differences in the repeated measures general linear model. Age, gender and education were used as covariates. ant = anterior; cent = central; gy = gyrus; inf = inferior; lat = lateral; mid = middle; orb = orbital; sup = superior.

ACE-R scores were positively correlated with PK11195 BP_ND_ in four regions, with the caudate showing the strongest correlation (R = 0.83, *P* = 0.00008). Significant positive correlations were also found between ACE-R scores and PK11195 BP_ND_ in the cuneus (R = 0.77, *P* = 0.0005), superior frontal gyrus (R = 0.69, *P* = 0.003) and anterior orbital gyrus (R = 0.67, *P* = 0.004). Whilst correlations with ACE-R scores in the remaining six regions were non-significant, all showed positive correlations, ranging from R = 0.25 to R = 0.63 (Fig. 2).

**Figure 3:**
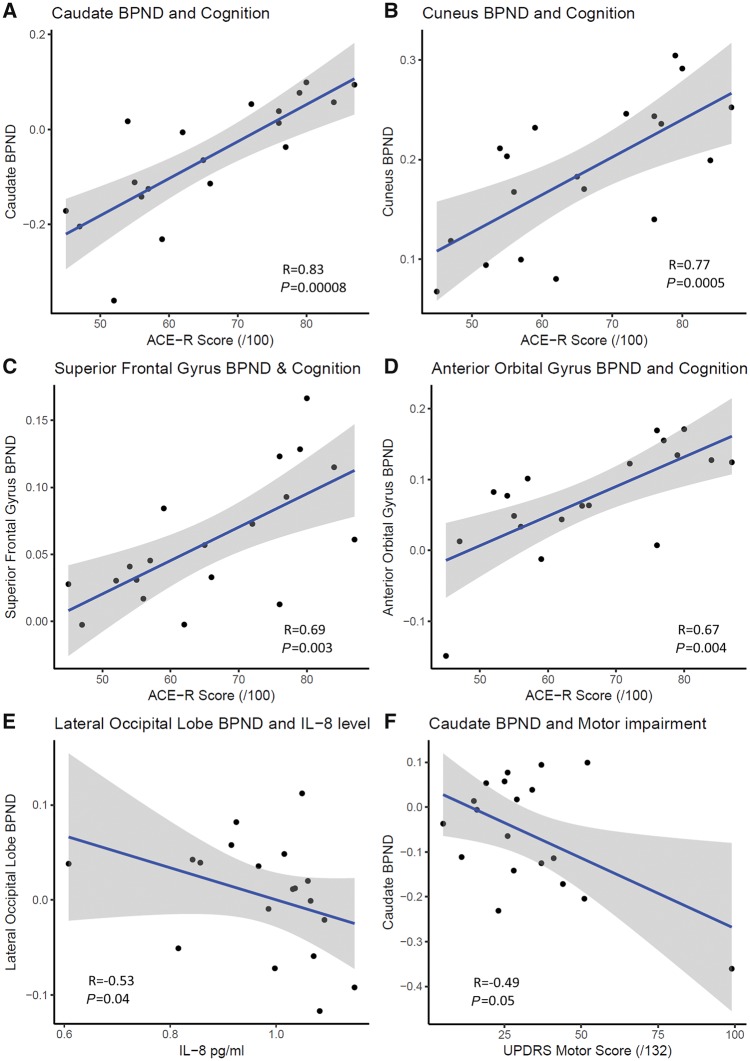
**Associations between clinical and inflammatory markers.** (**A**–**D**) Panels show strong positive associations between cognition and regional PK11195 binding. (**E**) Panel shows negative association between central inflammation in the occipital cortex and peripheral inflammation in the form of IL-8 levels in the blood. (**F**) Panel shows a negative association between the caudate PK11195 BP_ND_ and motor performance as measured by UPDRS. **A**–**D**, but not **E** and **F**, were statistically significant after correction for multiple comparisons.

There were also negative correlations between inflammatory cytokines IL-2 and IL-8 and PK11195 BP_ND_ in the lateral occipital lobe: R = −0.55, *P* = 0.03 and R = −0.53, *P* = 0.04, respectively, and between the caudate BP_ND_ and UPDRS scores (R = −0.49, *P* = 0.05), though these did not survive correction for multiple comparisons (Fig. 3).

Regional PK11195 binding did not correlate with PIB, either with cortical SUVR (Fig. 2) or with regional SUVR (Supplementary Table 3: PK11195 BPND and PIB SUVR regional correlations).

**Figure 4:**
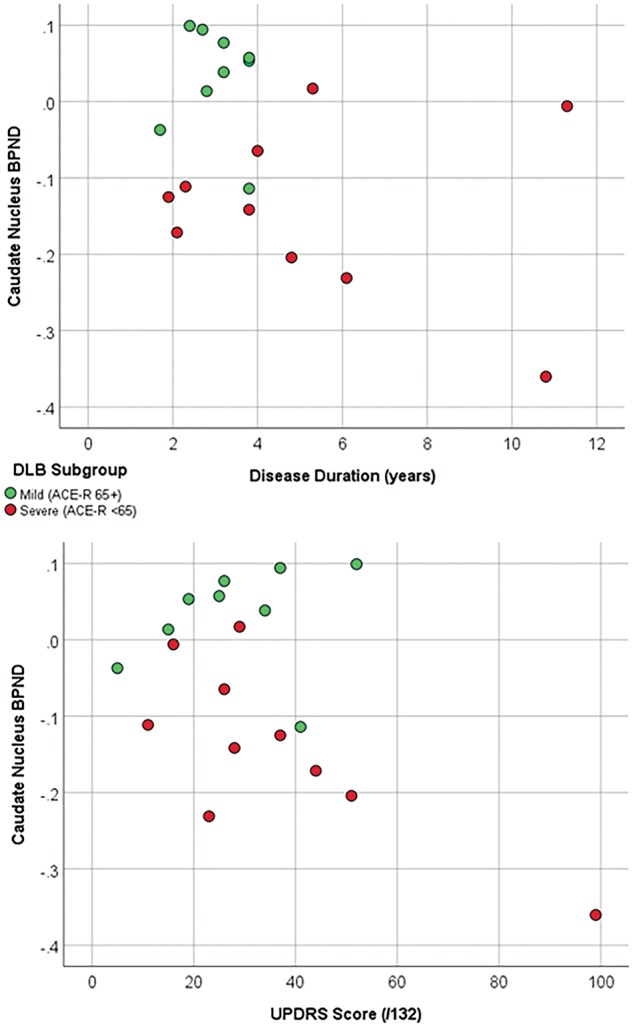
**Clinical features and caudate BP_ND_.** Comparison of caudate BP_ND_ with disease duration and UPDRS scores in the two DLB subgroups, suggesting that the association between microglial activation and cognition was independent of disease duration and motor impairment.

**Figure 5:**
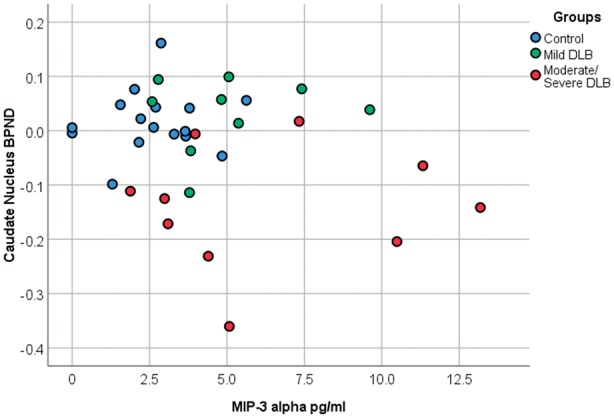
**Caudate BP_ND_ and MIP-3α.** Comparison of caudate BP_ND_ and MIP-3α levels in controls and the two DLB subgroups.

Comparison of PK11195 BP_ND_ in the caudate with disease duration and UPDRS scores appeared to show that higher binding was associated with the mild DLB group irrespective of disease duration and motor impairment (Fig. 4). Further comparison of PK11195 binding in the caudate region and levels of MIP-3α, showed that low levels of caudate binding and high levels of MIP-3α appeared to be associated with moderate/severe DLB (Fig. 5).

## Discussion

This study provides evidence of both central and peripheral inflammation in dementia with Lewy bodies and establishes their correlation with clinical severity.

PK11195 binding, which is a marker of microglial activation, was found to be significantly elevated in DLB cases with mild disease compared to those who had moderate/severe disease. Healthy adults had lower levels of inflammation than mild DLB cases, but higher levels than those with moderate/severe DLB, indicating a non-linear spectrum of microglial elevation, starting early in the condition and subsiding in the late stages. The strong association found between cognitive scores and microglial activation in several brain regions (caudate, cuneus, anterior orbital gyrus and superior frontal gyrus), as well as a positive correlation in all the regions tested, is consistent with such a continuum.

Early microglial activation is consistent with results in prodromal and early Alzheimer’s disease where PET studies have shown microglial activation in mild cognitive impairment, before the onset of dementia (Okello *et al.*, 2009) and early in dementia with a similar correlation with cognitive performance by means of MMSE score (Hamelin *et al.*, 2016). Inflammation has also been seen early in Parkinson’s disease in the brainstem, and cortical inflammation is seen before the onset of dementia (Ouchi *et al.*, 2005; Gerhard *et al.*, 2006; Edison *et al.*, 2013). In addition, inflammation has been reported even earlier in subjects with no motor or cognitive impairment, only REM sleep behaviour disorder, a condition that is recognized as a prodromal stage of synucleinopathies within the substantia nigra (Stokholm *et al.*, 2017) and occipital cortex (Stokholm *et al.*, 2018).

Early neuroinflammation that then plateaus has been reported in a mouse model of Alzheimer’s disease (López-Picón *et al.*, 2017), but to date a decline in microglial activation with disease progression has not been reported in human studies. Other studies in Alzheimer’s disease suggest an early and late peak (Fan *et al.*, 2017), though not demonstrated in the same subjects, or report an increase in inflammation with disease progression (Fan *et al.*, 2015; Hamelin *et al.*, 2018) but in only eight or six demented Alzheimer’s subjects, respectively, who individually had heterogeneous (both rises and falls in inflammation) on follow-up scanning. However, ^11^C-deuterium-l-deprenyl, which is a marker of astrogliosis, has been reported to be raised in PET imaging of patients with mild cognitive impairment but not established Alzheimer’s disease (Carter *et al.*, 2012), and also in autosomal dominant Alzheimer’s disease mutation carriers prior to the onset of dementia, before steadily declining despite concomitant increased PIB retention (Rodriguez-Vieitez *et al.*, 2016). In Parkinson’s disease, however, an inverse relationship between microglial activation and MMSE scores has also been found (Fan *et al.*, 2015), suggesting caution is indicated in the interpretation of this study’s result. Our study provides cross-sectional data only, and longitudinal studies are required to study the role of central inflammation as impairment progresses.

The strength of the association between cognition and inflammation was highest in the caudate, which was also identified by the SVM as a key classifier in determining differences between the control and DLB groups. The caudate is a core anatomical area involved in the pathology of DLB and caudate dysfunction could be caused by defects in the nigrostriatal pathway (Minoshima *et al.*, 2002) and/or their targets, the medium spiny neurons (Zaja-Milatovic *et al.*, 2006), both of which show selective degeneration in DLB, compared to Alzheimer’s disease. Early inflammation of the caudate, which declines with cognitive impairment, implicates a key component of the basal ganglia in the cognitive impairment seen in DLB. Indeed, hypometabolism in the caudate of DLB patients has previously been detected in early disease (Huang *et al.*, 2015). In addition, in a comparison with Parkinson’s disease patients, DLB patients are reported to have a more severe reduction in dopamine uptake within the caudate (Walker *et al.*, 2004; Gomperts *et al.*, 2016). The caudate has extensive cortical (as well as nigrostriatal) inputs and is increasingly recognized for its role in higher cognition, particularly executive function and goal-directed action (Grahn *et al.*, 2008; Haber, 2016).

A strong correlation between cognitive scores and PK11195 binding in the occipital lobe, within the cuneus, was also found. Occipital lobe pathology has been frequently reported in DLB, with both ^18^F-fluorodeoxyglucose (FDG) PET and perfusion single photon emission tomography (SPECT) scans showing reduced metabolism (Minoshima *et al.*, 2001; Kantarci *et al.*, 2012) and perfusion (Yeo *et al.*, 2013), respectively, distinguishing DLB cases from those with Alzheimer’s disease. Visuospatial dysfunction is also a specific indicator of DLB pathology (Yoshizawa *et al.*, 2013). Our results suggest inflammation may be linked to the underlying pathology of these impairments.

Furthermore, higher levels of microglial activation in the caudate appeared to be associated with milder cognitive impairment independently of disease duration or level of motor impairment, which may indicate a stronger link between caudate dysfunction and cognitive performance in DLB and a protective effect of microglial activation, at least initially. A longitudinal study in brain trauma patients found evidence of a protective role for microglia clinically—the drug minocycline reduced microglial activation over 12 weeks, with an associated increase in neurodegeneration (Scott *et al.*, 2018). However, early microglial activation that is then primed by systemic inflammatory factors towards chronic and deleterious inflammation has also been suggested as a potential mechanism in neurodegeneration (Perry and Holmes, 2014). A decline in microglial activation may be due to the conclusion of a deleterious process coinciding with the initiation of alternate pathways such as the triggering of the adaptive immune system, or may be due to exhaustion and dystrophic changes. Both have been reported in post-mortem studies in DLB (Mackenzie, 2000; Bachstetter *et al.*, 2015).

As well as central inflammation, we report increased peripheral cytokines in DLB. A significant cytokine × group interaction was found, suggesting individual cytokines had a different effect depending on the group that they were in. DLB participants showed higher levels of MIP-3α, IL-17A and IL-2 and lower levels of IL-8 in the serum compared to their healthy counterparts. MIP-3α, IL-2 and IL-8 were also identified by SVM as classifiers in separating the DLB group from controls. SVM was able to differentiate the groups with an accuracy of 81%, suggesting cytokine profiles between controls and DLB patients were indeed different.

MIP-3α, also known as CCL20, and IL-17A share a close relationship, with MIP-3α regulating helper T cells that produce IL-17a. IL-17a is strongly implicated in the pathogenesis of a number of autoimmune disorders. In rheumatoid arthritis in particular, IL-17a appears to promote a chronic pro-inflammatory state leading to bone and cartilage destruction (Schutyser *et al.*, 2003; Onishi and Gaffen, 2010; Lee and Körner, 2014) and levels have been found to fall following treatment of rheumatoid arthritis with monoclonal antibodies such as infliximab (Kawashiri *et al.*, 2009). Whether these two cytokines play a destructive inflammatory role in DLB requires further investigation.

IL-2 has a number of anti-inflammatory and pro-inflammatory roles within the immune system, but predominantly is a marker of T cell activation (Boyman and Sprent, 2012), again suggesting a role for T cells in DLB pathology. IL-8 is a mediator of inflammation through recruitment and degranulation of neutrophils, and can also promote phagocytosis in neutrophils (Waugh and Wilson, 2008).

Only one prior study has investigated peripheral cytokine levels in DLB (King *et al.*, 2017). No differences were found compared to healthy controls; however, MIP-3α, IL-17a and a large number of cytokines that we included in this current study were not investigated. IL-2 was not found to be raised in the DLB group but was raised in the prodromal DLB group and no differences in IL-8 results were found in either cohort. In Alzheimer’s disease, a systematic review of peripheral inflammatory markers showed IL-2 but not IL-8 was consistently raised (Lai *et al.*, 2017). MIP-3α and IL-17a were not mentioned in that review. Mouse models of Alzheimer’s disease, however, suggest T-helper cell infiltration into the brain parenchyma is combined with elevated IL-17 levels in the serum, CSF and hippocampus in association with amyloid pathology (Zhang *et al.*, 2013). T cells have also been implicated in the pathology of Alzheimer’s disease, through the IL-17 pathway (Sommer *et al.*, 2017).

Combined low levels of PK11195 binding in the caudate and higher levels of MIP-3α were associated with moderate/severe DLB and hint at a link between falling central inflammation and rising peripheral inflammation, involving the adaptive immune system. In addition, a negative, though non-significant, association was found between IL-2 and IL-8 levels and PK11195 binding in the occipital lobe. Rising systemic inflammation could be associated with a fall in central inflammation and further studies looking at this potential interaction are required.

We did not find any correlation between total cortical amyloid load and clinical features, regional PK11195 binding or peripheral cytokine levels. There were also no correlations found between amyloid load and PK11195 binding in the 10 regions where PK11195 binding showed the greatest significant differences in the general linear model, suggesting amyloid load is not a driver of inflammation or of disease in DLB, or that any such association is weak. This is despite 13 of 16 DLB participants who underwent PIB scans being classified as amyloid positive. A lack of correlation is however in contrast to other studies, which have shown a local or regional correlation between amyloid and inflammation in Parkinson’s disease dementia (Edison *et al.*, 2013) and Alzheimer’s disease (Fan *et al.*, 2015).

A possible limitation of the study is the lower specific binding of PK11195 for the translocator receptor (TSPO) compared to second generation ligands. However, unlike the second generation ligands, PK11195 is relatively unaffected by the genetic polymorphisms of TSPO that lead to high, low and mixed affinity binders (Owen *et al.*, 2012), especially between high and mixed affinity binders (Guo *et al.*, 2012; Kobayashi *et al.*, 2018) that represent at least 90% of the Caucasian population and form even higher proportions in other populations (Owen *et al.*, 2012). In addition, all reports so far show no significant difference in binding of PK11195 in the CNS between the three genotypes (Fujita *et al.*, 2017).

Overall, our results suggest DLB is associated with early microglial activation in key areas affected by DLB pathology, which declines as cognitive impairment progresses. Peripherally, cytokines associated with T cell activation appear to be higher in DLB. The next step is for a longitudinal study of central and peripheral inflammation in early DLB, to understand if progressive disease is linked to both pathways, and hence if selectively targeting either could halt disease progression.

## Supplementary Material

Supplementary Data

## Abbreviations

ACE-R: Addenbrooke’s Cognitive Examination-Revised
BP_ND_: non-displaceable binding potential
DLB: dementia with Lewy bodies
IL: interleukin
MIP-3α: macrophage inflammatory protein 3α
MMSE: Mini-Mental State Examination
PIB: Pittsburgh compound B
SUVR: standardized uptake value ratio
SVM: support vector machine
UPDRS: Unified Parkinson’s Disease Rating Scale part III (motor)
