# Supplementary material for: Early microglial activation and peripheral inflammation in dementia with Lewy bodies
Source: Brain. 2018 Nov 6;141(12):3415–27. doi: 10.1093/brain/awy265 (PMC6262214; doi:10.1093/brain/awy265)
Supplement: Supplementary Data [file awy265_suppl_data.pdf]

Cytokine	LLOQ	Missing Data
hsCRP mg/L	0.20	0
IL-34 pg/ml	30	0
YKL40 pg/ml	3125	0
MCSF1 pg/ml	78	0
TNFR1 (CD120a) pg/ml	39	0
IL- 17A Gen. B pg/ml	3	0
IL-21 pg/ml	1.65	0
IL-22 pg/ml	2.78	0
IL-23 pg/ml	4.55	0
IL-27 pg/ml	38.7	0
IL-31 pg/ml	4.22	0
MIP-3a pg/ml	0.588	0
GM-CSF pg/ml	1.90	0
IL-1α pg/ml	2.85	0
IL-12 pg/ml	5.68	0
IL-15 pg/ml	1.4	0
IL-16 pg/ml	19.1	0
IL-17A pg/ml	9.32	1 DLB
IL-5 pg/ml	6.28	0
IL-7 pg/ml	1.37	0
TNF-β pg/ml	1.15	0
VEGF pg/ml	7.7	0
Eotaxin pg/ml	12.3	0
Eotaxin 3 pg/ml	10.2	0
IP10 pg/ml	1.37	0
MCP-1 pg/ml	1.09	0
MCP-4 pg/ml	5.13	0
MDC pg/ml	88.3	0
MIP1a pg/ml	13.8	0
MIP1b pg/ml	2.27	0
TARC pg/ml	3.32	0
IFN gamma pg/ml	7.47	0
IL-10 pg/ml	0.680	0
IL-12p70 pg/ml	1.22	1 Control
IL-13 pg/ml	4.21	0
IL-1β pg/ml	2.14	0
IL-2 pg/ml	0.890	0
IL-4 pg/ml	0.450	0
IL-6 pg/ml	1.58	0
IL-8 pg/ml	1.13	0
TNF α pg/ml	0.690	0

Supplementary Table 1: Details of Cytokine Assays

LLOQ = Lower Limit of Quanitification

	Mild DLB (n=9)	Severe DLB (n=10)	Control Group – PET Imaging (n=16)	Group Difference (Mild DLB v Control - PET)	Group Difference (Severe DLB v Control – PET)	Group Difference (Mild DLB v Severe DLB)
Gender (males/females)	6/3	9/1	8/8	*P*=0.68	*P*=0.09	*P*=0.30
Age in years: mean (± SD)	74.7(± 5.2)	71.5(± 6.7)	70.0 (± 6.5)	t=1.87; *P*=0.07	t=0.55; *P*=0.59	t=1.17; *P*=0.26
Education in years: mean (± SD)	11.8(± 1.9)	11.7(± 2.1)	14.1(± 3.0)	t=-2.2; *P*=0.04	t=-2.3; *P*=0.03	t=0.09; *P*=0.93
MMSE scores: mean (± SD)	25.9(± 2.7)	18.3(± 1.8)	28.9(± 1.1)	t=-4.1; *P*<0.001	t=17.2; *P*<0.001	t=7.4; *P*<0.001
ACE-R scores: mean (± SD)	77.4(± 6.2)	55.2(± 6.2)	92.5(± 5.6)	t=-6.2; *P*<0.001	t=15.9; *P*<0.001	t=7.8; *P*<0.001
UPDRS scores: mean (± SD)	28.2(±14.4)	36.4(±25.1)	N/A	N/A	N/A	t=-0.86; *P*=0.40
Disease duration in years: mean (± SD)	3.0(±0.7)	5.2(±3.4)	N/A	N/A	N/A	t=-1.9; *P*=0.07
^{11}C-PK11195 PET scan	9	10	16			
^{11}C-PiB PET scan	9	7	0			

Supplementary Table 2: Demographics - Early, Mild, Control PET Group comparison

The Mild and Severe DLB groups were matched with the control group, as well as each other, for gender and age. There were differences in education between the Mild and Severe DLB groups and the controls but not each other. As expected, MMSE and ACE-R scores were significantly different between each of the three groups. Disease duration and UPDRS scores were however not significantly different between Mild and Severe DLB participants.

**Region**	**Pearson's R**	**P value**
Caudate	0.14	0.65
Inferior Frontal Gyrus	-0.23	0.46
Cuneus	-0.44	0.14
Lateral Occiptal Lobe	-0.39	0.19
Middle and Inferior Temporal Gyrus	-0.24	0.42
Superior Central Temporal Gyrus	-0.22	0.47
Lateral Orbital Gyrus	-0.02	0.95
Fusiform Gyrus	0.09	0.77
Anterior Orbital Gyrus	0.11	0.71
Superior Frontal Gyrus	-0.19	0.53

Supplementary Table 3: PK11195 BPND and PiB SUVR Regional Correlations. Pearson's partial correlations with age, gender and education as covariates
